# Kindness Shown in Suffering

**Published:** 1890-07-26

**Authors:** 


### Words of Consolation.

### KINDNESS SHOWN IN SUFFERING.

"How can I, who am fast bound by weakness and suffering myself, be kind to anyone?" you say; "surely it is the duty of other people to be kind to me?" Undoubtedly it is so, but if we look at the subject a little more closely we shall see that it is also the duty of the feeblest invalids to show this grace in their actions. For what is kindness? Simply the doing to others as we should like them to do to us. It has the power of making the world comparatively happy, indeed of causing it to be quite a different world to what it is now.

Kindness is the "overflowing of self upon others;" we put others in the place of self, and so we fulfil our dear Lord's commands. What greater anguish can wring the heart than seeing those we love suffer either in body or mind, without our being able to relieve them? This inability to help made the sword so sharp which pierced the Virgin Mother's heart as she stood beneath the Cross of Calvary and saw her Son with bleeding hands and feet, the victim of untold agonies. We have never witnessed anything so dreadful, and therefore cannot feel as she did; neither have we stood beside a death bed of a divine son whose life had been sinless as His. Yet let us think for a few moments on the pain our sufferings caused to those who see them, and learn from our Blessed Master to be kind in suffering.

He, while hanging on the Cross, spoke three times to those who were mourning and grieving over His agonies, for He must needs help and comfort all who mourned with Him. With readiness and loving kindness He replied to the prayer of the penitent thief and rewarded his faith with the promise of Paradise. Again, He commended His Blessed Mother to the care of the beloved disciple, and thus left us an example to make provision if possible for our relations when death draws near. Was not this to suffer kindly, and is it not a lesson to us who are often so selfish in our woe?

What must the Centurion have felt who had directed, and the rough soldiers, who had with cruel hands scourged and crucified the Lord of Glory, when the terrible darkness came over the earth, which trembled and quaked beneath their feet? The patience of the Lord during these fearful sights and sounds must surely have melted their hearts, while in the midst of their bitter repentance and abject terror and despair, came the pitying, loving, extenuating prayer, "Father forgive them, for they know not what they do." Behold and see if there be any sorrow like unto His sorrow, or any depths of love so great as His! And what can we do, dear Lord, to be like Thee? We may pity and forgive those who are grieving because they have injured us and caused us pain. A blow, perhaps, has been struck in sudden anger which may have crippled or blinded us for life. We feel as if we could never forgive the culprit, although he is bowed down with sorrow at the misery he has caused and which he cannot undo. It is a hard thing for us to accomplish; the natural temper revolts against it, but do not the words echo in our hearts, "Forgive them for they knew not what they did"? Should they not rest there and make us speak gently, as did our Great Pattern, till we can pray to God in the sinner's behalf and try to win his soul?

Another way to be kind as our Lord was, is to have sympathy with those who are in pain like ourselves, to cheer them if we have the opportunity, to remember them in our prayers. Sick people are frequently sadly wanting in sympathy with each other. When they are told of others' ailments they try to make out they are better off than themselves, their circumstances are better, or they are more tenderly nursed, or they have not so much to trouble them and aggravate their malady. Let us then try instead to imitate our dear Lord by thinking of other people before ourselves.

